# *In vivo* structural modification of type II arabinogalactans with fungal endo-β-1, 6-galactanase in Arabidopsis

**DOI:** 10.3389/fpls.2022.1010492

**Published:** 2022-11-09

**Authors:** Aina Kikuchi, Katsuya Hara, Yoshihisa Yoshimi, Kouichi Soga, Daisuke Takahashi, Toshihisa Kotake

**Affiliations:** ^1^ Division of Life Science, Graduate School of Science and Engineering, Saitama University, Saitama, Japan; ^2^ Department of Biochemistry, University of Cambridge, Cambridge, United Kingdom; ^3^ Department of Biology, Graduate School of Science, Osaka Metropolitan University, Osaka, Japan; ^4^ Green Bioscience Research Center, Saitama University, Saitama, Japan

**Keywords:** cellulose synthesis, cell wall, endo-β-1,6-galactanase, *in vivo* modification, type II arabinogalactan

## Abstract

Arabinogalactan-proteins (AGPs) are mysterious extracellular glycoproteins in plants. Although AGPs are highly conserved, their molecular functions remain obscure. The physiological importance of AGPs has been extensively demonstrated with β-Yariv reagent, which specifically binds to AGPs and upon introduction into cells, causes various deleterious effects including growth inhibition and programmed cell death. However, structural features of AGPs that determine their functions have not been identified with β-Yariv reagent. It is known that AGPs are decorated with large type II arabinogalactans (AGs), which are necessary for their functions. Type II AGs consist of a β-1,3-galactan main chain and β-1,6-galactan side chains with auxiliary sugar residues such as L-arabinose and 4-*O*-methyl-glucuronic acid. While most side chains are short, long side chains such as β-1,6-galactohexaose (β-1,6-Gal_6_) also exist in type II AGs. To gain insight into the structures important for AGP functions, *in vivo* structural modification of β-1,6-galactan side chains was performed in Arabidopsis. We generated transgenic Arabidopsis plants expressing a fungal endo-β-1,6-galactanase, Tv6GAL, that degrades long side chains specifically under the control of dexamethasone (Dex). Two of 6 transgenic lines obtained showed more than 40 times activity of endo-β-1,6-galactanase when treated with Dex. Structural analysis indicated that long side chains such as β-1,6-Gal_5_ and β-1,6-Gal_6_ were significantly reduced compared to wild-type plants. Tv6GAL induction caused retarded growth of seedlings, which had a reduced amount of cellulose in cell walls. These results suggest that long β-1,6-galactan side chains are necessary for normal cellulose synthesis and/or deposition as their defect affects cell growth in plants.

## Introduction

Plant cell walls are composed of various polysaccharides including cellulose, pectic polysaccharides, and hemicelluloses (Mohnen, 2008). Among them, type II arabinogalactan (AG) is one of the most complex polysaccharides present in the cell wall and on the plasma membrane (Seifert and Roberts, 2007). Because of its complex and heterogenous carbohydrate structure, molecular functions of type II AGs are unclear and remain to be elucidated.

**Figure 1:**
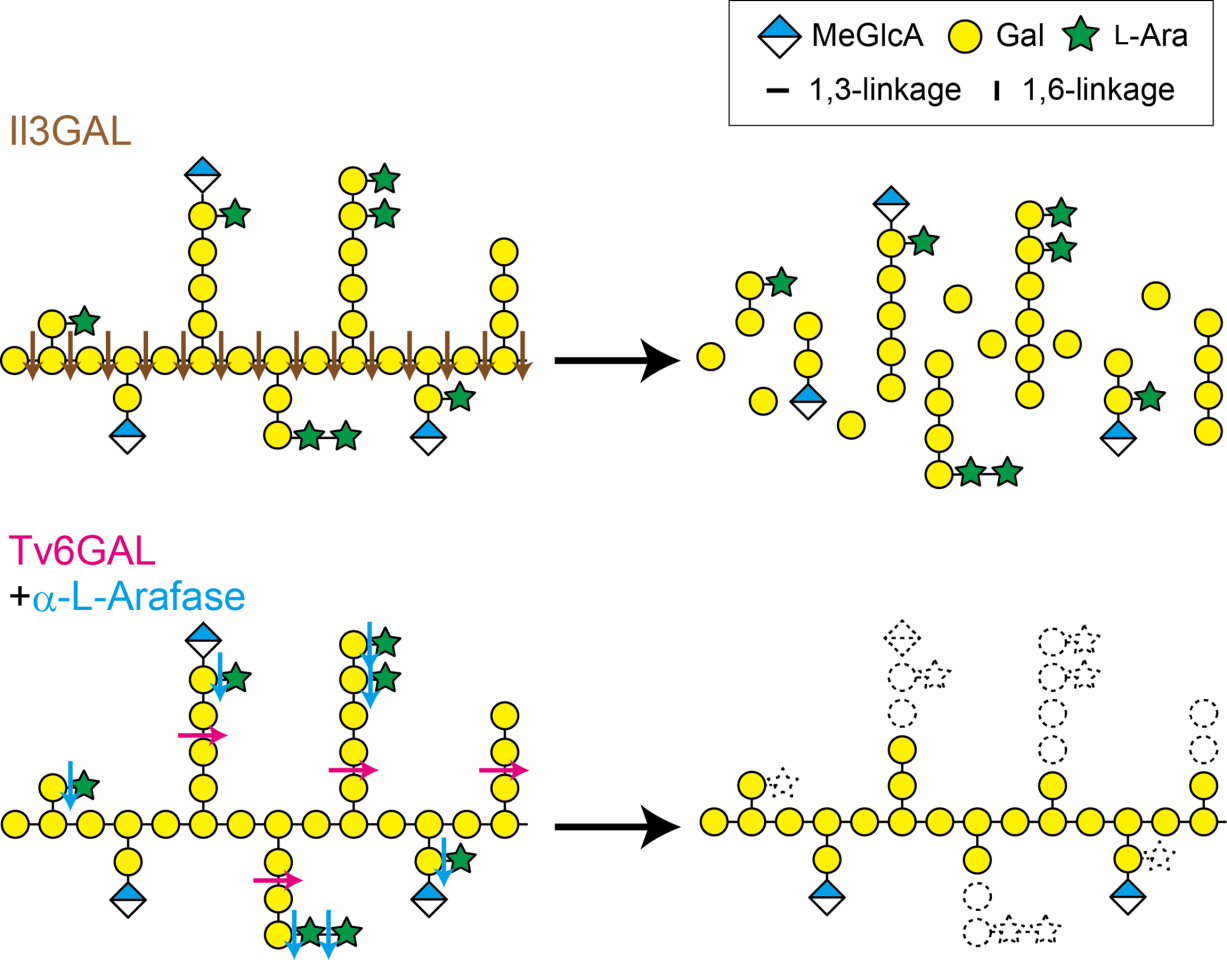
Il3GAL and Tv6GAL act differently on type II AG. Il3GAL specifically hydrolyzes the β-1,3-galactan main chain irrespective of the presence of β-1,6-galactan side chains. As a result of the action of Il3GAL, type II AG is degraded into oligosaccharides. Tv6GAL specifically hydrolyzes long β-1,6-galactan side chains of type II AG longer than β-1,6-Gal_2_, leaving only short side chains.

Type II AGs are generally found as carbohydrate moieties of arabinogalactan-proteins (AGPs) that are conserved extracellular glycoproteins in plants (Fincher et al., 1983; Seifert and Roberts, 2007; Ma et al., 2018). Type II AGs are necessary for AGP functions. For example, a non-classical type of AGP, called xylogen, loses its activity to induce the differentiation of tracheary elements in zinnia (*Zinnia elegans*) cells by chemical deglycosylation (Motose et al., 2004). Generally, the basic structure of type II AG is β-1,3:1,6-galactan, in which β-1,6-galactan side chains are attached onto a β-1,3-galactan main chain (**Figure 1**). The β-1,6-galactan side chains are further decorated with L-arabinose (Ara) and other auxiliary sugars such as glucuronic acid (GlcA), 4-*O*-methyl-GlcA (MeGlcA), L-fucose (Fuc), and L-rhamnose (Rha). (Tan et al., 2010; Tryfona et al., 2010; Tryfona et al., 2012; Shimoda et al., 2014; Inaba et al., 2015). Although the structure of type II AG, in particular auxiliary sugars, is diverse, depending on plant species, tissues, and developmental stages, several common features can be found in the β-1,3:1,6-galactan (Tsumuraya et al., 1987; Tsumuraya et al., 1988). First, the substitution ratio of β-1,3-galactan with β-1,6-galactosyl side chains in type II AGs ranges from 46 to 58% in many angiosperms (Ito et al., 2020). Second, β-1,6-galactose (β-1,6-Gal) is the dominant side chain, but long side chains such as β-1,6-galactohexaose (β-1,6-Gal_6_) also occur as minor side chains in type II AGs (Ito et al., 2020).

The physiological importance of AGPs has been extensively demonstrated with β-Yariv reagent that specifically binds to the β-1,3-galactan main chain of type II AG (Yariv et al., 1967a; Yariv et al., 1967b; Seifert and Roberts, 2007; Kitazawa et al., 2013). For example, the treatment with β-Yariv reagent results in disorganization and depolymerization of cortical microtubules, probably the reason for cell bulging in tobacco suspension-cultured cells (Sardar et al., 2006). However, since the binding is to β-1,3-galactan main chains irrespective of other carbohydrate structures (Kitazawa et al., 2013), it is not obvious how to identify other important carbohydrate structures through studies with β-Yariv reagent. Moreover, β-Yariv reagent may cause secondary and side effects by forming insoluble precipitates with AGPs (Olmos et al., 2017).

The carbohydrate structure of type II AG is primarily determined during its synthesis. In Arabidopsis, many glycosyltransferases (GTs) involved in the synthesis of type II AG have been identified (Silva et al., 2020). Among them, GALT29A (At1g08280) is presumed to have a function in the attachment of β-1,6-galactosyl residues onto the β-1,3-galactan main chain in Arabidopsis (Dilokpimol et al., 2014). However, the expected structural changes in β-1,6-galactan side chains in type II AGs have not been observed in the loss-of-function mutant.

The carbohydrate structures are also changed through enzymatic degradation. Type II AGs undergo hydrolysis by endogenous glycoside hydrolases (GHs). Plant cells secret β-galactosidase, α-L-arabinofuranosidase (α-L-Arafase), β-L-arabinopyranosidase, and β-glucuronidase (β-GlcAase) to hydrolyze type II AGs (Kotake et al., 2005; Kotake et al., 2006; Eudes et al., 2008; Imaizumi et al., 2017). Because these plant GHs act only on non-reducing terminal residues, they do not drastically change type II AG structures. On the other hand, GHs secreted by fungi and bacteria hydrolyze type II AGs more efficiently than plant GHs. An exo-β-1,3-galactanase from *Irpex lacteus*, Il3GAL, hydrolyzes the β-1,3-galactan main chain bypassing β-1,6-galactan side chains, causing the degradation of type II AGs into oligosaccharides (Tsumuraya et al., 1990; Ichinose et al., 2005; Kotake et al., 2009; Matsuyama et al., 2020). An endo-β-1,6-galactanase from *Trichoderma viride* (*Hypocrea rufa*), Tv6GAL, specifically hydrolyzes β-1,6-galactan side chains in an endo-manner (**Figure 1**). The enzyme can specifically remove long β-1,6-galactan side chains from β-1,3:1,6-galactan, leaving only stubs of short side chains such as β-1,6-Gal on the main chain (Okemoto et al., 2003; Kotake et al., 2004) (**Figure 1**). Thus, these specific fungal GHs are reliable tools for structural and functional analysis of type II AGs (Kitazawa et al., 2013; Yoshimi et al., 2020).

We have recently developed a novel system to study type II AGs in Arabidopsis, in which type II AGs are specifically hydrolyzed into oligosaccharides by an exo-β-1,3-galactanase, Il3GAL, *in vivo* (Yoshimi et al., 2020). In *Dex::Il3GAL* plants, we have observed severe tissue disorganization caused by expression of the *Il3GAL* gene in hypocotyls and cotyledons, demonstrating the importance of type II AGs in the regulation of cell shape. On the other hand, we could not examine the importance of β-1,6-galactan side chains in type II AGs, as Il3GAL hydrolyzed type II AGs into oligosaccharides in this system. For the present study, we thus developed another system in which long β-1,6-galactan side chains are trimmed by the fungal endo-β-1,6-galactanase Tv6GAL in Arabidopsis. In the transgenic Arabidopsis, we observed high endo-β-1,6-galactanase activity and a significant decrease in long β-1,6-galactan side chains. Based on the retarded growth phenotype and changes in cell wall fractions of the transgenic plants, we discuss the physiological importance and involvement of long β-1,6-galactan side chains in cellulose synthesis and deposition.

## Materials and methods

### Transgenic Arabidopsis

Arabidopsis (*Arabidopsis thaliana*) ecotype Col-0 was used in this study. To generate transgenic Arabidopsis expressing *Tv6GAL* under the control of Dex, the Dex-inducible expression system with a binary vector, the pTA7001 plasmid, was used (Aoyama and Chua, 1997). The cDNA of *Tv6GAL* without signal sequence was amplified by PCR with specific primers Tv6GAL-F+BamHI and Tv6GAL-R+XbaI and cloned into pGEM-T-Easy vector (Promega) (**Supplementary Table S1**). For the generation of point-mutated Tv6GAL (Tv6GAL-PM), the mutation E210A replacing a putative catalytic residue Glu with Ala was introduced by PCR with a set of specific primers (**Supplementary Figure S1**; **Supplementary Table S1**). This residue is conserved for GH30 family enzymes including Tv6GAL. *Tv6GAL* and *Tv6GAL-PM* fragments were fused with a signal sequence of Arabidopsis *AGP4* amplified with AGP4signal+XhoI and AGP4signal-R+BamHI on pTA7001 (**Supplementary Table S1**) (Aoyama and Chua, 1997; Yoshimi et al., 2020). To generate *Dex::Tv6GAL* and *Dex::Tv6GAL-PM* plants, the plasmid constructs were introduced into Arabidopsis by Agrobacterium (*Rhizobium radiobacter*) mediated transformation (Clough and Bent, 1998). *Dex::Il3GAL* plants have been generated in our previous study (Yoshimi et al., 2020).

Plants were grown on Murashige-Skoog (MS)-agar media (Murashige and Skoog, 1962) in the presence of 10 μM Dex dissolved in DMSO. As the negative control for the treatment, the plants were treated with an equal amount of pure DMSO. For the assay of endo-β-1,6-galactanase activity of transgenic plants, the seeds were first incubated in the dark at 4°C for 2 days and grown under continuous light at 23°C for 2 weeks, whereas for the measurement of hypocotyl length, they were grown in the dark at 23°C for 5 days. Hypocotyl length was measured using ImageJ software.

### Preparation of recombinant Tv6GAL protein

*Tv6GAL* and *Tv6GAL-PM* fragments without signal sequence were amplified by PCR and subcloned into the restriction sites EcoRI and XbaI of pPICZαC plasmid (Invitrogen, Waltham, MA, USA) as described previously (Takata et al., 2010). The methylotrophic yeast *Pichia pastoris* strain KM71 (Invitrogen) was then transformed by electroporation with the pPICZαC plasmids. The transformed *Pichia* yeasts were cultured in YPG medium containing 1% (w/v) yeast extract, 2% (w/v) peptone, and 1% (w/v) glycerol at 28°C with shaking at 90 rpm for 48 h. The cells were collected by centrifugation at 1,500 *g* for 5 min, washed with ice-cold water, and suspended in 50 ml of YP medium containing 1% (w/v) yeast extract and 2% (w/v) peptone. To induce the expression of recombinant Tv6GAL (rTv6GAL) or recombinant Tv6GAL-PM (rTv6GAL-PM), the yeast was cultured for another 4 days with addition of 1% (v/v) methanol each day. The rTv6GAL and rTv6GAL-PM proteins were collected in the supernatant of the culture medium by centrifugation at 1,500 *g* for 5 min and purified on a SP-Toyopearl HF 40F column (Tosoh, Tokyo, Japan) as described previously (Takata et al., 2010). The purity of the recombinant enzymes was confirmed by SDS–PAGE (Laemmli, 1970).

### Measurement of the mechanical properties of hypocotyls

Seedlings grown in the dark for 5 days were boiled for 10 min in 80% (v/v) ethanol and then stored in fresh 80% ethanol until analysis. Before measuring the mechanical properties of cell walls, seedlings fixed in 80% ethanol were rehydrated with several changes of water. The cell wall extensibility and the breaking load of the middle region of hypocotyls were measured using a tensile tester (Tensilon STB-1225S; A&D Co. Ltd., Tokyo, Japan). Hypocotyls were fixed between two clamps (distance between clamps was 1 mm) and stretched by raising the upper clamp at a speed of 20 mm/min until the hypocotyls broke. The cell wall extensibility (strain/load in units of μm/g) was determined by measuring the load’s rate of increase from 1 to 2 g.

### Determination of endo-β-1,6-galactanase activity

Endo-β-1,6-galactanase activity of rTv6GAL and rTv6GAL-PM was measured with a reaction mixture containing 0.5 mg/mL of algal β-1,6-galactan prepared from *Prototheca zopfii* that has long β-1,6-galactans together with short β-1,3-galactans (Okemoto et al., 2003), 50 mM sodium acetate buffer (pH 5.0) and enzyme at 37°C. The reducing sugars were measured colourimetrically by the Neocuproine method (Dygert et al., 1965). One unit of enzyme activity is capable of producing sugars equivalent to one µmol of glucose (Glc) from the substrate per minute.

To measure the activity of *Dex::Tv6GAL* and *Dex::Tv6GAL-PM* plants, the seedlings grown in the presence of 10 μM Dex were homogenized in 20 mM sodium acetate buffer (pH 5.0) containing 1 M NaCl with mortar and pestle. The homogenate was centrifuged at 10,000 *g*, and the resulting supernatant was collected and used as enzyme solution. The protein concentration was determined by the method of Bradford with bovine serum albumin as the standard (Bradford, 1976).

### Measurement of cellulose content

The cellulose content of transgenic Arabidopsis was determined by using modified Updegraff method (Foster et al., 2010; Dampanaboina et al., 2021). The seedlings of transgenic Arabidopsis grown with 10 µM Dex under continuous light for 10 days were homogenized and treated with 50 mM Tris-HCl (pH 8.8), 0.5 mM ethylenediaminetetraacetic acid (EDTA), and 10% (w/v) SDS at 25°C. After washing by centrifugation with same buffer once and water twice, the precipitate was suspended in 70% (v/v) ethanol and heated at 70°C for 1 h. The precipitate was further washed with 70% (v/v) ethanol once, methanol once, and methanol/chloroform (1:1, v/v) once and divided into two fractions for the determination of total polysaccharides and cellulose. The fraction for cellulose was washed with acetone once and dried up. The polysaccharides other than cellulose in this fraction was hydrolyzed by the Updegraff reagent (73% (v/v) acetic acid and 9% (v/v) nitric acid) at 100°C for 30 min and removed. The remaining cellulose was washed with water twice, ethanol once, and diethyl ether once and dried up. These fractions were hydrolyzed with 72% (v/v) sulfuric acid at 25°C for 1 h and then 8% (v/v) sulfuric acid at 100°C for 4 h. The sugar amount of the fractions was measured by the phenol-sulfuric acid method (Dubois et al., 1956). The proportion of non-cellulosic polysaccharides and cellulose was calculated.

### Structural analysis of type II AGs

Structural analysis by specific fragmentation with enzymes was performed as described (Ito et al., 2020). Type II AGs in the soluble fraction were dialyzed to remove mono- and oligosaccharides and hydrolyzed into Gal, L-Ara, MeGlcA, and β-1,6-galactooligosaccharides with exo-β-1,3-galactanase Il3GAL, endo-β-1,3-galactanase from *Flammulina velutipes*, β-GlcAase from *Aspergillus niger*, and α-L-Arafase from *Aspergillus niger* (Megazyme, Wicklow, Ireland) at 37°C for three hours (**Supplementary Figure S2**) (Konishi et al., 2008; Kotake et al., 2009; Yoshimi et al., 2017). The liberated β-1,6-galactooligosaccharides and monosaccharides were derivatized with *p*-aminobenzoic acid ethyl ester (ABEE) (Matsuura and Imaoka, 1988), and detected by an HPLC system as described previously (Ito et al., 2020; Yoshimi et al., 2020). Note that β-1,6-galactooligosaccharides liberated in this reaction respectively have one Gal residue that derives from the β-1,3-galactan main chain.

## Results

### *In vitro* modification of β-1,3:1,6-galactan

**Figure 2:**
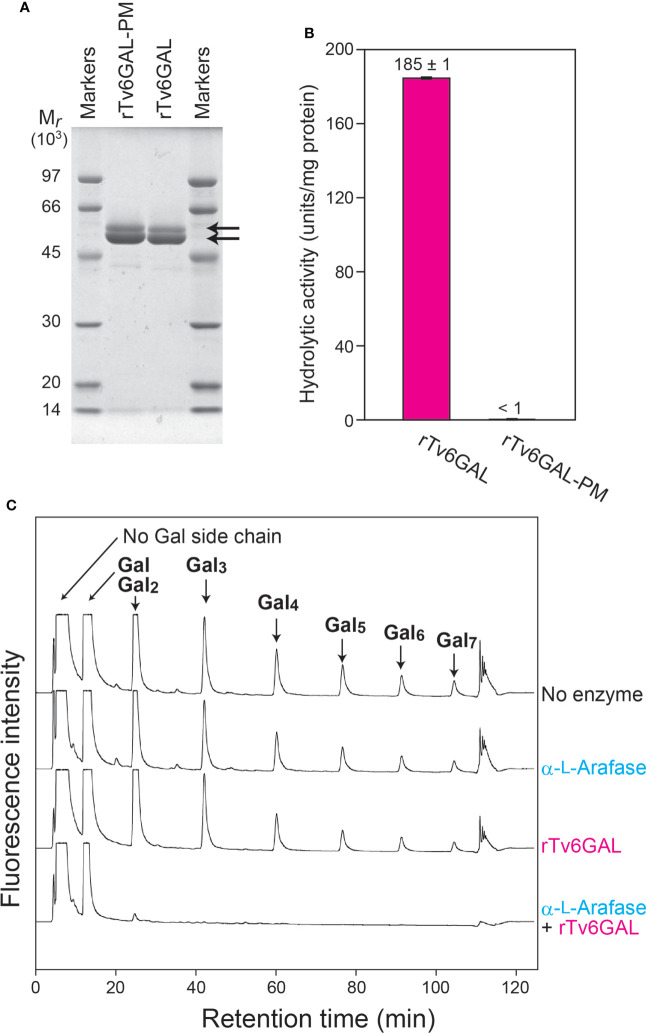
*In vitro* structural modification of β-1,3:1,6-galactan. **(A)** The expression of rTv6GAL and rTv6GAL-PM in *P. pastoris*. The purified rTv6GAL and rTv6GAL-PM were analyzed on SDS-PAGE. Proteins in the gel were stained with Coomassie Brilliant Blue R-250. Arrows indicate rTv6GAL and rTv6GAL-PM. **(B)** Hydrolytic activity of rTv6GAL and rTv6GAL-PM toward algal β-1,6-galactan. **(C)** Change in the length of β-1,6-galactan side chains. β-1,3:1,6-Galactan reacted with rTv6GAL was hydrolyzed into β-1,6-galactooligosaccharides using Il3GAL, NcEn3GAL, AnGlcAase, and α-L-Arafase (**Supplementary Figure S2**). The resulting mono- and oligosaccharides were derivatized with ABEE and analyzed on HPLC. Note that each β-1,6-galactooligosaccharide has one Gal derived from the β-1,3-galactan main chain. For example, a β-1,6-Gal_2_ side chain is detected as 1,6-Gal_3_ in this analysis. M*_r_*, relative molecular mass.

The endo-β-1,6-galactanase Tv6GAL had been identified and cloned from *T. viride* (*H. rufa*) in our previous study (Okemoto et al., 2003; Kotake et al., 2004). It differs from plant β-galactosidases in that it specifically acts on β-1,6-galactan in an endo-manner. For the present study, this specific action was confirmed *in vitro* using recombinant proteins. The rTv6GAL and rTv6GAL-PM were expressed in *Pichia* yeast and purified (**Supplementary Figure S1**). These recombinant proteins appeared as two protein bands with relative molecular masses of 56 and 51 kDa on SDS-PAGE, whereas the calculated molecular weight for mature Tv6GAL is 50,589 (**Figure 2A**). It is presumed that differential *N*-glycosylation on these recombinant proteins in *Pichia* yeast is responsible for the difference (Gemmill and Trimble, 1999; Kotake et al., 2009; Strasser, 2016). The unmutated rTv6GAL showed strong hydrolytic activity (185 units/mg protein) on algal β-1,6-galactan used as substrate, but rTv6GAL-PM had extremely low activity (less than 1 unit/mg protein) (**Figure 2B**).

It has been shown that the removal of Ara residues remarkably enhances the hydrolysis of type II AG by Tv6GAL (Tsumuraya et al., 1988; Kotake et al., 2004). Here, we examined the hydrolysis of radish root AGP (Tsumuraya et al., 1988) by rTv6GAL alone, and by rTv6GAL combined with α-L-Arafase. The enzymatically modified AGPs were purified by size-exclusion chromatography to remove released mono- and oligosaccharides. Then, the product AGPs were subjected to structural analysis focusing on the length of β-1,6-galactan side chains. In this analysis, the type II AG was specifically hydrolyzed into β-1,6-galactooligosaccharides by combinational hydrolysis with Il3GAL, endo-β-1,3-galactanase (NcEn3GAL), β-GlcAase, and an α-L-Arafase (Konishi et al., 2008; Kotake et al., 2009; Kotake et al., 2011; Ito et al., 2020) (**Supplementary Figure S2**). The oligosaccharides were labeled with fluorescence and analyzed on HPLC. As shown in **Figure 2C**, the type II AG completely lost side chains longer than β-1,6-Gal_3_ by combinational hydrolysis of rTv6GAL and α-L-Arafase: note that a β-1,6-Gal_3_ side chain is detected as β-1,6-Gal_4_ in this analysis as all oligosaccharides detected here have one Gal residue derived from the β-1,3-galactan main chain (**Supplementary Figure S2**). On the other hand, rTv6GAL alone did not efficiently act on β-1,6-galactan side chains. These results suggest that Tv6GAL can hydrolyze long β-1,6-galactan side chains of type II AG in combination with endogenous α-L-Arafase *in vivo*.

### Transgenic Arabidopsis expressing fungal endo-β-1,6-galactanase

We generated transgenic Arabidopsis, *Dex::Tv6GAL* plants that express the *Tv6GAL* gene under the control of Dex-inducible promoter. For proper secretion of Tv6GAL, the original signal peptide of Tv6GAL was replaced with that from Arabidopsis AGP4 (AtAGP4) as had been done for *Dex::Il3GAL* plants (Yoshimi et al., 2020). We obtained six lines (#1, #3, #5, #6, #8, and #12) of *Dex::Tv6GAL* plants and two lines (#1 and #2) of *Dex::Tv6GAL-PM* plants as the negative control. The transgenic plants were grown in the presence of Dex to induce the expression of *Tv6GAL* or *Tv6GAL-PM* gene under continuous light.

**Figure 3:**
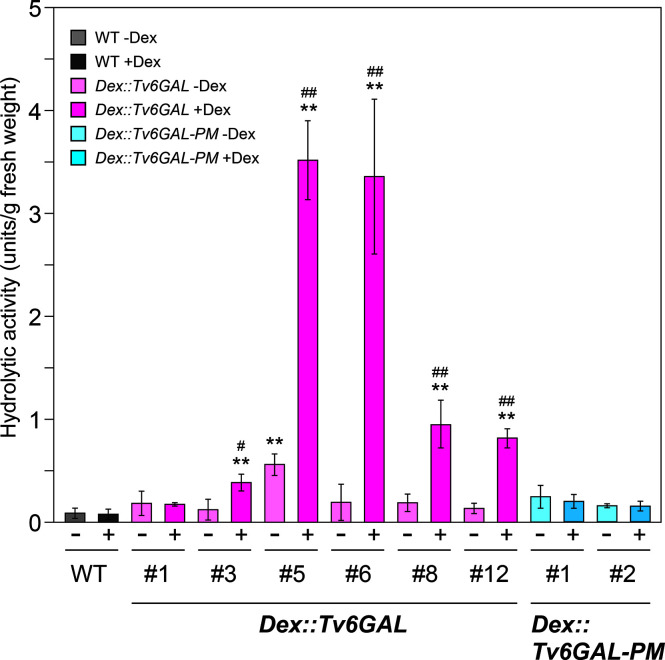
Enzymatic activity of *Dex::Tv6GAL* and *Dex::Tv6GAL-PM* plants. Endo-β-1,6-galactanase activity was measured using algal β-1,6-galactan as substrate. Endo-β-1,6-galactanase activity per g fresh weight is shown. Data are mean values with ± SD (n=3 biological replicates). Asterisks indicate differences from WT plants with Dex treatment (Student’s *t*-test, **, *P*<0.01) and hash marks indicate significant differences between seedlings with and without Dex treatment (Student’s *t*-test, #, *P*<0.05; ##, *P*<0.01).

To evaluate the transgenic lines, we first measured endo-β-1,6-galactanase activity of transgenic Arabidopsis using algal β-1,6-galactan as the substrate (Okemoto et al., 2003). Out of six lines of *Dex::Tv6GAL* plants, five lines (#3, #5, #6, #8, and #12) showed significantly higher activity (0.38, 3,52, 3,36, 0.95, and 0.82 units/g fresh weight) than wild-type (WT) Arabidopsis (0.08 units/g fresh weight) when treated with Dex (**Figure 3**). On the other hand, the activity of *Dex::Tv6GAL-PM* plants was comparable to WT plants. This indicates that Tv6GAL is properly synthesized in Arabidopsis and functions as endo-β-1,6-galactanase in this system. *Dex::Tv6GAL* plants #5 and #6 exhibited remarkably higher activity than the others, therefore these lines were mainly used in the following experiments. In line #5, leaky expression of *Tv6GAL* likely occurred without Dex treatment. Weak activity detected in WT plants is presumed to be endogenous β-galactosidase activity, as plant β-galactosidases also hydrolyze this algal β-1,6-galactan (Kotake et al., 2005).

### *In vivo* modification of type II AGs

**Figure 4:**
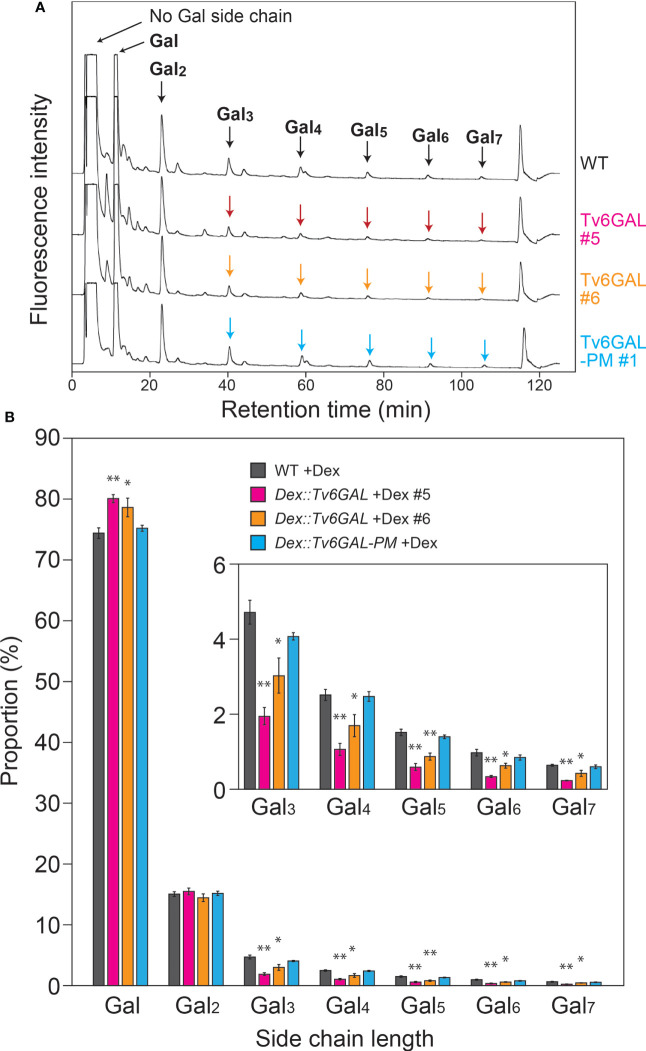
*In vivo* structural modification of type II AGs. **(A)** Representative chromatogram of β-1,6-galactooligosaccharides. Type II AGs were extracted from WT, *Dex::Tv6GAL* #5 and #6, and *Dex::Tv6GAL-PM* #1 plants grown in the presence of Dex and subjected to structural analysis as performed for *in vitro* modification to obtain the distribution of chain lengths of β-1,6-galactooligosaccharides (**Supplementary Figure S2**). In this chromatogram, β-1,6-galactooligosaccharides larger than β-1,6-Gal_2_ were examined. **(B)** Distribution of Gal lengths of β-1,6-galactan side chains. The distribution of β-1,6-galactan side chains was calculated based on the peak areas of β-1,6-galactooligosaccharides. Data are mean values with ± SD (n=3 biological replicates). Asterisks indicate significant differences from WT plants (Student’s *t*-test, *, *P*<0.05; **, *P*<0.01).

The main purpose of this study was to investigate the structural changes in β-1,6-galactan side chains of type II AGs *in vivo*. We did not express α-L-Arafase with Tv6GAL, because plants secret type II AG-active α-L-Arafases in cell walls (Kotake et al., 2006). To examine the *in vivo* structural change, type II AGs were extracted from light-grown *Dex::Tv6GAL* plants #5 and #6 and subjected to specific hydrolysis into β-1,6-galactooligosaccharides by a combination of enzymes followed by labeling with fluorescence and HPLC analysis as performed for the analysis of *in vitro* modification of type II AGs. Consistent with other plants such as spinach (*Spinacia oleracea*) and broccoli (*Brassica oleracea* var. italica) examined in a previous study (Ito et al., 2020), short side chains such as β-1,6-Gal and β-1,6-Gal_2_ were very dominant over longer side chains including β-1,6-Gal_5_ and β-1,6-Gal_6_ in WT Arabidopsis (**Figure 4A**). Compared with WT plants, Dex-treated *Dex::Tv6GAL* plants #5 and #6 had a significantly reduced proportion of long β-1,6-galactan side chains such as β-1,6-Gal_5_ and β-1,6-Gal_6_ (**Figure 4B**). Consistent with endo-β-1,6-galactanase activity, Dex treatment did not significantly change β-1,6-galactan side chains in *Dex::Tv6GAL-PM* plants. In this study, β-1,6-galactan side chains longer than 1,6-Gal_7_ could not be analyzed due to technical limits, however it is expected that these very long side chains were also hydrolyzed in the lines #5 and #6. Indeed, our previous study has identified β-1,6-Gal_10_ and β-1,6-Gal_15_ in type II AGs (Shimoda et al., 2014; Tsumuraya et al., 2019). Approximately half of the β-1,6-Gal_5_ and β-1,6-Gal_6_ remained in these lines treated with Dex. This may be caused by differential accumulation of Tv6GAL in the tissues and/or the decoration with other sugar residues that will be explained in the Discussion.

### Retarded growth of *Dex::Tv6GAL* plants

**Figure 5:**
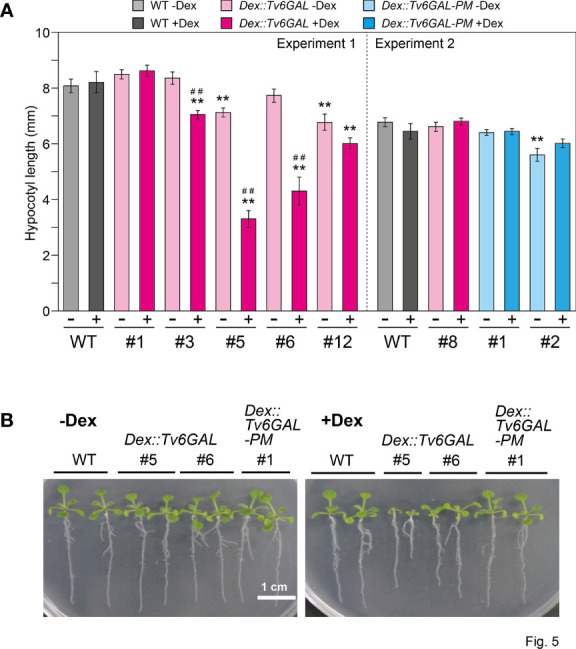
Phenotypes of *Dex::Tv6GAL* plants. **(A)** Length of dark-grown hypocotyls. *Dex::Tv6GAL* and *Dex::Tv6GAL-PM* plants were grown on MS-agar plates with and without 10 µM Dex in the dark at 23°C for 5 days. The data show mean values with ± SD. n = 20. Asterisks indicate significant differences from WT plants (Student’s *t*-test, **, *P*<0.01) and hash marks indicate significant differences between seedlings with and without Dex treatment (Student’s *t*-test, ##, *P*<0.01). Representative plants are shown in **Supplementary Figure S3**. **(B)** Light-grown seedlings. The seedlings were grown on MS-agar plates with and without 10 µM Dex under continuous light at 23°C for 2 weeks. Representative plants were selected.

The physiological importance of AGPs in growth and development has been shown in Arabidopsis mutants and by treatment of plants with β-Yariv reagent (Silva et al., 2020; Kaur et al., 2021). In many cases, the perturbation of AGP functions results in reduced growth. In our previous study, *Dex::Il3GAL* Arabidopsis expressing the *Il3GAL* gene exhibited severe tissue disorganization with extremely bulged epidermal cells in light-grown seedlings and growth inhibition in dark-grown hypocotyls, demonstrating the physiological importance of type II AGs (Yoshimi et al., 2020). In this study, the influence of hydrolysis of long β-1,6-galactan side chains by Tv6GAL on the elongation growth of dark-grown hypocotyls was examined. Out of five lines of *Dex::Tv6GAL* plants, four lines (#3, #5, #6, and #12) exhibited significantly shorter hypocotyls (9, 60, 48, and 27% decrease, respectively) under Dex treatment than WT plants (**Figure 5A**). Consistent with endo-β-1,6-galactanase activity, the growth inhibition was more significant in lines #5 and #6 than in the other lines (**Supplementary Figure S3**). Growth inhibition was not observed in *Dex::Tv6GAL-PM* plants, although one line not treated with Dex had shorter hypocotyls than WT plants. The phenotypes of light-grown seedlings were also analyzed. Apparent dwarfism was seen in *Dex::Tv6GAL* plants #5 in the presence of Dex, while the growth phenotypes of line #6 were relatively weak compared with line #5 (**Figure 5B**). In addition, severe phenotypes occurred in *Dex::Il3GAL* plants including epidermal cell bulging and severe tissue disorganization were not observed in *Dex::Tv6GAL* plants.

**Figure 6:**
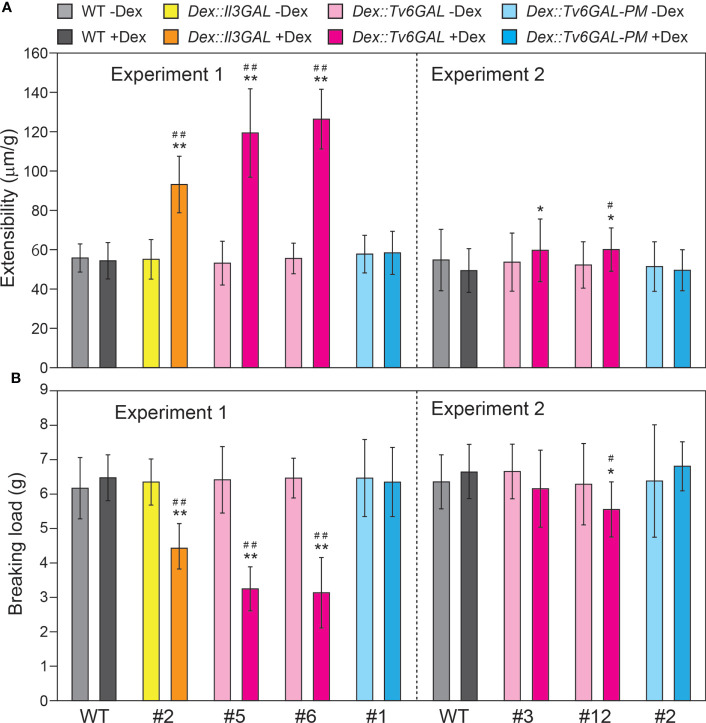
Mechanical properties of *Dex::Tv6GAL* plants. **(A)** Extensibility of hypocotyls. **(B)** Breaking load. The plants were grown on MS-agar plates with and without 10 µM Dex in the dark at 23°C for 5 days. The mechanical properties of the middle region of hypocotyls were measured by a tensile test. The data show mean values with ± SD, n = 20. Asterisks indicate significant differences from WT plants (Student’s *t*-test, *, *P*<0.05; **, *P*<0.01) and hash marks indicate significant differences between plants with and without Dex treatment (Student’s *t*-test, #, *P*<0.05; ##, *P*<0.01).

The mechanical properties of dark-grown hypocotyls were determined by a tensile test. Together with *Dex::Il3GAL* plants #2, *Dex::Tv6GAL* plants #5, #6, and #12 showed significantly higher extensibility and lower breaking load when they were grown in the presence of Dex (**Figure 6**). On the other hand, *Dex::Tv6GAL-PM* plants #1 and #2 had properties comparable to those of WT plants. The changes in the mechanical properties may be the result of altered cell wall components and structure as well as tissue organization.

### Decreased cellulose in *Dex::Tv6GAL* plants

The retarded growth and altered mechanical properties of *Dex::Tv6GAL* plants suggest that the decrease in long β-1,6-galactan side chains in type II AGs affected cell wall synthesis, modification, and/or metabolism. To investigate the influence of Tv6GAL expression on cellulose synthesis and deposition, we measured the cellulose content by the Updegraff method (Foster et al., 2010; Dampanaboina et al., 2021). The lines #5 and #6 of *Dex::Tv6GAL* plants grown in the presence of Dex had lower proportion of cellulose in total polysaccharides (25 and 31%, respectively) than WT (42%), indicating that type II AGs are necessary for proper cellulose synthesis and/or deposition. The changes in the lines #1 of *Dex::Tv6GAL-PM* plants were not observed. The reduced proportion of cellulose in the line #5 of *Dex::Tv6GAL* plants was also confirmed by cell wall fractionation into hot water (HW), EDTA, alkali, and cellulose fractions (**Supplementary Figure S4**). The line #5 showed a significant decrease in cellulose fraction and an increase in HW and EDTA fractions. Sugar composition analysis revealed that the line #5 had an increased proportion of Glc and a decreased proportion of GalA in HW fraction (**Supplementary Table S2**-**S4**). Interestingly, decreased cellulose and increased Glc in other fractions were observed for *Dex::Il3GAL* plants (**Supplementary Figure S4**), and have also been reported in the Arabidopsis temperature-sensitive *radial swelling 2* (*rsw2*, allelic to *korrigan*) mutant with a defect in cellulose synthesis (Lane et al., 2001).

## Discussion

### *In vivo* modification of β-1,6-galactan side chains

Type II AGs are the second most complex carbohydrates after pectin rhamnogalacturonan II in plants, as they have a heterogeneous β-1,3:1,6-galactan backbone, to which Ara, GlcA, MeGlcA, and other auxiliary sugars are attached. Type II AGs are conserved polysaccharides that can generally be found as glycan parts of AGPs in plants. Due to their heterogenicity and complexity, the identification of specific structures important for the functions of type II AGs is not easy. GHs acting on specific structures are reliable tools in the study of complex carbohydrates. Here we used a fungal endo-β-1,6-galactanase Tv6GAL to change β-1,6-galactan *in vivo* and observed a significant reduction of long β-1,6-galactan side chains in two lines of *Dex::Tv6GAL* plants for the first time (**Figure 4**). In these transgenic plants, the long side chains are presumably changed to short side chains such as β-1,6-Gal and β-1,6-Gal_2_ by Tv6GAL. On the other hand, this *in vivo* hydrolysis of type II AGs was far from complete compared with *in vitro* modification of β-1,3:1,6-galactan. We suggest several possible reasons. First, there may be β-1,6-galactan side chains highly substituted with Ara residues that are resistant to Tv6GAL (Shimoda et al., 2014), although plants have high endogenous α-L-Arafase activity (Kotake et al., 2006). Tv6GAL requires removal of α-L-arabinofuranosyl residues for efficient hydrolysis of type II AGs (Kotake et al., 2004) (**Figure 2**). The presence of β-1,6-galactan highly substituted with Ara residues should be examined by using specific antibodies such as KM1 in a future study (Pfeifer et al., 2022). Second, not all tissues may have expressed the *Tv6GAL* gene at a high level because, for the expression of *Tv6GAL*, not only permeation by Dex but also accumulation in the same cell of the glucocorticoid receptor-fused transcription activation domain, which is transcribed from the transgene, is necessary. It is quite probable that the accumulation of Tv6GAL differed by tissues and developmental stages. Third, β-1,6-galactan side chains of type II AGs might be masked by the interaction with other cell wall components *in vivo*.

### Functions of type II AGs in cellulose synthesis

**Figure 7:**
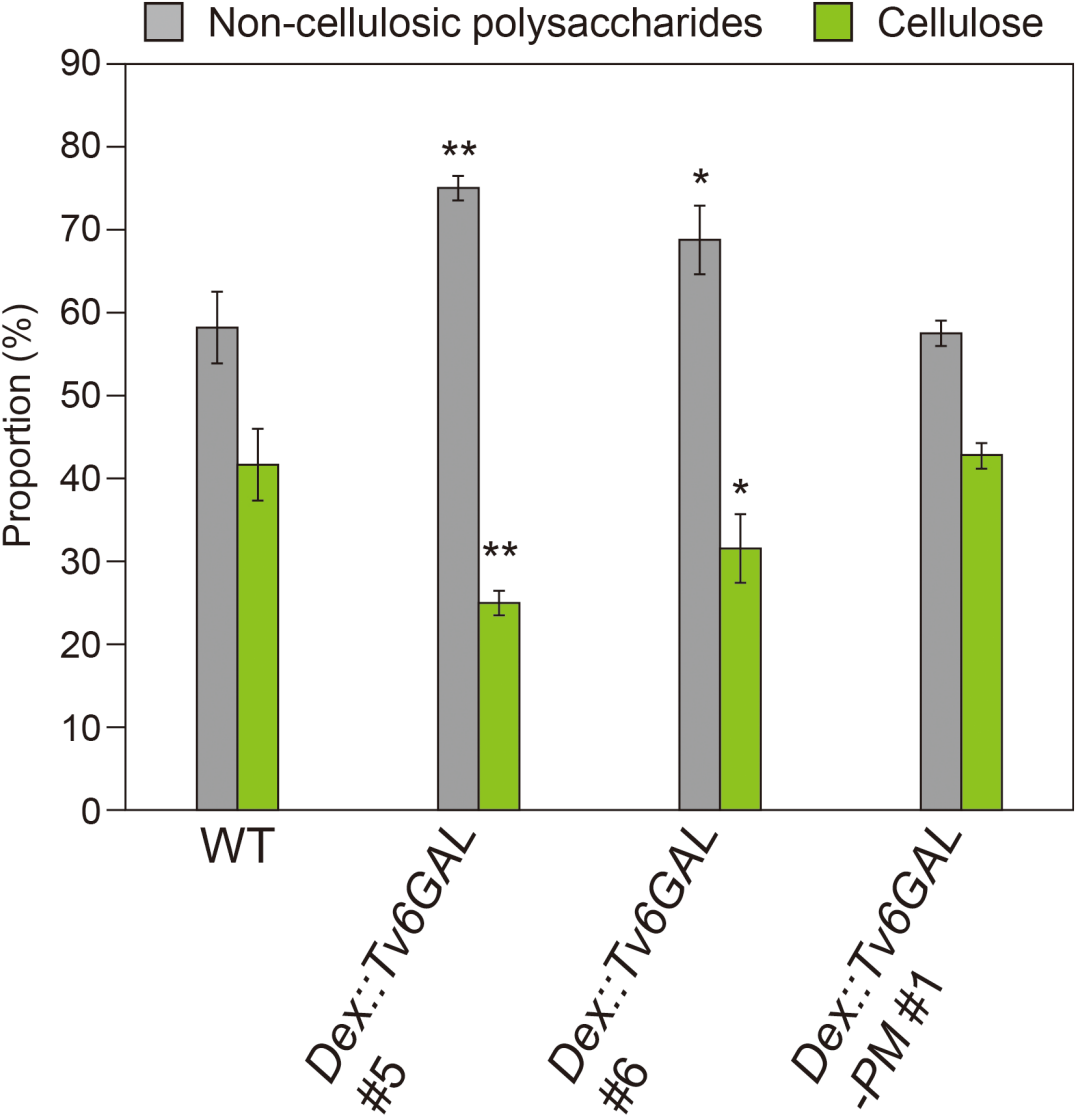
Proportion of cell wall fractions. Plants were grown on MS-agar media containing 10 µM Dex under continuous light for 10 days. Using the Updegraff method, the proportion of non-cellulosic polysaccharides and cellulose was determined. Data are mean values with ± SD (n = 3 biological replicates). Asterisks indicate significant differences from WT plants (Stud’s *t*-test, *, *P*<0.05; **, *P*<0.01).

For the functional analysis of type II AGs, Arabidopsis mutants with a defect in the synthesis of type II AGs are useful. The synthesis of type II AGs starts with Gal transfer from UDP-Gal onto hydroxyproline by the action of HPGT-GALT proteins. Then the chain elongation of the β-1,3-galactan main chain by β-1,3-galactosyltransferases, called CAGEs, occurs (Basu et al., 2013; Ogawa-Ohnishi and Matsubayashi, 2015; Nibbering et al., 2022). Importantly, an Arabidopsis *cage1 cage2* double mutant has been shown to have a significantly reduced amount of crystalline cellulose in shortened dark-grown hypocotyls (Nibbering et al., 2022). Because the protein levels of cellulose synthase catalytic subunits, CesAs, were significantly reduced in the double mutant while their expression levels were not changed, type II AGs are presumed to regulate CesAs by a post-transcriptional or post-translational mechanism. In addition, an Arabidopsis quintuple mutant of HPGT-GALTs also showed stunted growth and root swelling, which reminds us of the phenotypes of cellulose-deficient Arabidopsis mutants such as *cobra* and *korrigan* (Kaur et al., 2021). In the present study, the significantly reduced amount of cellulose fraction observed for *Dex::Tv6GAL*, as well as *Dex::Il3GAL* plants (**Figure 7**), suggests the participation of long β-1,6-galactan side chains in cellulose synthesis and/or deposition.

A recent study on Arabidopsis fasciclin-like AGP 11 (FLA11) and FLA12 has proposed that they perform a sensing function in the regulation of secondary cell wall synthesis in response to mechanical stress (Ma et al., 2022). In contrast with the studies on the *cage1 cage2* double mutant, the over-expression of the *FLA11* gene greatly affected the expression of CesA genes. Supporting the suggestion of a regulatory function of FLAs at the expression level, a drastic decrease in the expression level of the *CesA8* gene has also been observed in the Arabidopsis *fla16* mutant (Liu et al., 2020). Thus, molecular functions of AGPs in cellulose synthesis are still elusive, but it is highly probable that type II AGs including long β-1,6-galactan side chains have molecular functions different from those of core-protein domains such as the fasciclin-like domain.

No severe tissue disorganization was observed in *Dex::Tv6GAL* plants (**Figure 5**), which contrasts with *Dex::Il3GAL* plants (Yoshimi et al., 2020). In addition, the growth phenotypes of light-grown seedlings were weaker than those of *Dex::Il3GAL* plants. At this moment, we do not have a clear explanation for these differences. Given that Tv6GAL caused reduced function of long β-1,6-galactan side chains while Il3GAL perturbed the functions of type II AGs by releasing various β-1,6-galactan side chains from type II AG, severe tissue disorganization may occur only by the perturbation of type II AG functions. The phenotypes of these plants will be further analyzed in a future study.

### Important carbohydrate structures for functions of type II AGs

For different physiological functions, different carbohydrate structures of type II AGs are required, although type II AGs are relatively minor components in cell walls. Indeed, GlcA and MeGlcA residues have been shown to be important for the Ca^2+^ capacitor function of AGPs through a study on Arabidopsis mutants defective in β-glucuronidation of type II AGs (Lopez-Hernandez et al., 2020). In Torenia (*Torenia fournieri*), MeGlcA-Gal was identified as an important structure for the signaling function of type II AGs in pollen tube guidance (Mizukami et al., 2016). This structure, called AMOR, makes pollen tubes responsive to the attractant peptide LURE. Interestingly, the methyl group of MeGlcA is indispensable for the function of AMOR. In the present study, we have identified long β-1,6-galactan side chains as important structures for the participation in cellulose synthesis or deposition. Through this function, long β-1,6-galactan side chains are presumed to affect cell elongation.

Our previous study has shown that the proportion of β-1,6-galactan side chains with a given Gal length is probably conserved in angiosperm. Consistent with five other plant species examined in that study (Ito et al., 2020), here the proportions of β-1,6-Gal_5_ and β-1,6-Gal_6_ were less than 2% each in Arabidopsis seedlings. These observations suggest that the long stretches of β-1,6-galactan occurring as minor side chains are conserved beyond plant species and physiologically important. Side chains much longer than β-1,6-Gal_7_ likely exist in Arabidopsis. However, they must be very rare structures, as Gal and β-1,6-Gal_2_ side chains did not largely increase when these very long side chains were converted to short side chains by Tv6GAL in *Dex::Tv6GAL* plants. However, we cannot exclude the possibility that the important β-1,6-galactan side chains are those that are much longer than β-1,6-Gal_5_ and β-1,6-Gal_6_. We note in this context that larch AG and gum arabic that lacks long side chains will not participate in cellulose synthesis.

## Conclusion

In the present study, *in vivo* structural modification of long β-1,6-galactan side chains of type II AGs was performed for the first time. The phenotypes of transgenic plants expressing an endo-β-1,6-galactanase Tv6GAL suggested the importance of long β-1,6-galactan side chains and the involvement of type II AGs in cellulose synthesis or deposition. The present study also suggests that different carbohydrate structures are required for different physiological functions of type II AGs.

## Data availability statement

The original contributions presented in the study are included in the article/**Supplementary Material**. Further inquiries can be directed to the corresponding author.

## Author contributions

YY and TK designed the research. AK, KH, YY, KS, and TK performed the experiments and data collection. AK, KH, KS, DT, and TK analyzed the data. YY, KS, DT, and TK wrote the manuscript. All authors approved the final version of manuscript.

## Funding

This research was supported by MEXT KAKENHI Grant-in-Aid for Scientific Research on Innovative Areas “Plant-Structure Optimization Strategy” to TK (no. 18H05495), by Grants-in-Aid for Scientific Research to TK (no. 19K06702) and to DT (no. 20K15494), and by a Broodbank Research Fellowship to YY (no. PD16178).

## Acknowledgments

We thank Prof. Emeritus Yoichi Tsumuraya for technical advice and valuable discussion. We are also grateful to Mr. Tatsuya Kutsuno and Mr. Keita Higuchi for technical assistance.

## Conflict of interest

The authors declare that the research was conducted in the absence of any commercial or financial relationships that could be construed as a potential conflict of interest.

## Publisher’s note

All claims expressed in this article are solely those of the authors and do not necessarily represent those of their affiliated organizations, or those of the publisher, the editors and the reviewers. Any product that may be evaluated in this article, or claim that may be made by its manufacturer, is not guaranteed or endorsed by the publisher.
