# Supplementary material for: In vivo structural modification of type II arabinogalactans with fungal endo-β-1, 6-galactanase in Arabidopsis
Source: Front Plant Sci. 2022 Nov 9;13:1010492. doi: 10.3389/fpls.2022.1010492 (PMC9682044; doi:10.3389/fpls.2022.1010492)
Supplement: Supplementary file 1 [file DataSheet_1.docx]

Supplementary Table S1. List of primers used in this study.

The restriction sites are shown in italic font.

| **Primer** | **Sequence (5’ to 3’)** |
| --- | --- |
| AGP4signal-F+*Xho*I | 5’-*CTCGAG*ATTATGGGTTCCAAGATTG-3’ |
| AGP4signal-R+*BamH*I | 5’-*GGATCC*AGCGAGTGCTGAAGTGGCGA-3’ |
| Tv6GAL-F+*Bam*HI | 5’-*GGATCC*ATGGACACCACGCTTACCATC-3’ |
| Tv6GAL-R+*Xba*I | 5’-C*TCTAGA*GCTCATTGCAACACAACG-3’ |
| Tv6GAL-PM-F | 5’-CGTCTTCCAGCTGGTGGAC-3’ |
| Tv6GAL-PM-R | 5’-GCGCGTTGAATGCCTCAACTG-3’ |
| Tv6GAL-F+*Bam*HI | 5’-*GAATTC*ATGGACACCACGCTTACCATC-3’ |

Supplementary Table S2. Sugar composition of the HW fractions.

Polysaccharides were dialyzed against water for 2 days, hydrolyzed with 2 M trifluoroacetic acid at 121°C for one hour, and applied to HPEAC-PAD to determine the sugar composition. Rha, rhamnose; Man, mannose; Xyl, xylose; GalA, galacturonic acid. Data are mean values with ±SD (*n* = 3 biological replicates). Asterisks indicate significant differences from WT plants (Student’s *t*-test, *, *P*<0.05; **, *P*<0.01).

|  |  |  |  | Composition (mol%) | | | | | |  |  |  |
| --- | --- | --- | --- | --- | --- | --- | --- | --- | --- | --- | --- | --- |
|  | WT | | |  | *Dex::Il3GAL* #2 | | |  | *Dex::Tv6GAL* #5 | | |  |
| Fuc | 1.5 | ± | 0.2 |  | 1.9 | ± | 0.5 |  | 1.0 | ± | 0.2 |  |
| Rha | 4.6 | ± | 0.7 |  | 2.8 | ± | 0.5 |  | 1.8 | ± | 0.3 | * |
| Ara | 12.0 | ± | 1.0 |  | 9.6 | ± | 0.5 | * | 6.1 | ± | 0.8 | ** |
| Gal | 14.1 | ± | 2.0 |  | 11.0 | ± | 0.6 |  | 8.9 | ± | 3.0 |  |
| Glc | 34.6 | ± | 3.3 |  | 59.1 | ± | 2.7 | ** | 66.8 | ± | 4.1 | ** |
| Man | 4.0 | ± | 0.3 |  | 3.6 | ± | 0.2 |  | 2.7 | ± | 0.3 | * |
| Xyl | 5.7 | ± | 1.9 |  | 4.0 | ± | 0.2 |  | 2.4 | ± | 0.4 |  |
| GalA | 22.8 | ± | 1.3 |  | 8.0 | ± | 0.6 | ** | 9.9 | ± | 2.6 | ** |
| GlcA | 0.8 | ± | 0.5 |  | 0.1 | ± | 0.0 |  | 0.4 | ± | 0.3 |  |

Supplementary Table S3. Sugar composition of the EDTA fractions.

Data are mean values with ±SD (*n* = 3 biological replicates). Asterisks indicate significant differences from WT plants (Student’s *t*-test, *, *P*<0.05; **, *P*<0.01).

|  |  |  |  | Composition (mol%) | | | | | |  |  |  |
| --- | --- | --- | --- | --- | --- | --- | --- | --- | --- | --- | --- | --- |
|  | WT | | |  | *Dex::Il3GAL* #2 | | |  | *Dex::Tv6GAL* #5 | | |  |
| Fuc | 2.1 | ± | 0.3 |  | 1.8 | ± | 0.2 |  | 1.5 | ± | 0.4 |  |
| Rha | 13.2 | ± | 0.4 |  | 12.1 | ± | 1.7 |  | 13.4 | ± | 1.3 |  |
| Ara | 18.0 | ± | 0.4 |  | 15.5 | ± | 1.4 |  | 15.4 | ± | 0.9 | * |
| Gal | 16.0 | ± | 1.5 |  | 12.3 | ± | 1.5 |  | 11.2 | ± | 0.9 | * |
| Glc | 0.5 | ± | 0.1 |  | 0.3 | ± | 0.1 |  | 0.3 | ± | 0.1 |  |
| Man | 0.2 | ± | 0.1 |  | 0.3 | ± | 0.0 |  | 0.3 | ± | 0.1 |  |
| Xyl | 4.3 | ± | 0.4 |  | 3.0 | ± | 0.7 |  | 2.5 | ± | 0.5 | * |
| GalA | 44.7 | ± | 1.5 |  | 53.7 | ± | 2.1 | * | 54.5 | ± | 1.5 | ** |
| GlcA | 1.0 | ± | 0.0 |  | 1.0 | ± | 0.0 |  | 0.9 | ± | 0.1 |  |

Supplementary Table S4. Sugar composition of the alkali fractions.

Data are mean values with ±SD (*n* = 3 biological replicates). Asterisks indicate significant differences from WT plants (Student’s *t*-test, *, *P*<0.05; **, *P*<0.01).

|  |  |  |  | Composition (mol%) | | | | | |  |  |  |
| --- | --- | --- | --- | --- | --- | --- | --- | --- | --- | --- | --- | --- |
|  | WT | | |  | *Dex::Il3GAL* #2 | | |  | *Dex::Tv6GAL* #5 | | |  |
| Fuc | 5.2 | ± | 0.1 |  | 4.2 | ± | 0.6 |  | 4.3 | ± | 0.1 | ** |
| Rha | 7.3 | ± | 0.3 |  | 8.7 | ± | 0.3 | ** | 8.3 | ± | 0.5 |  |
| Ara | 17.4 | ± | 1.1 |  | 21.8 | ± | 2.8 |  | 19.2 | ± | 2.8 |  |
| Gal | 22.2 | ± | 1.9 |  | 22.0 | ± | 2.6 |  | 20.3 | ± | 2.0 |  |
| Glc | 20.6 | ± | 1.4 |  | 15.2 | ± | 3.1 |  | 18.7 | ± | 4.0 |  |
| Man | 1.6 | ± | 0.3 |  | 1.8 | ± | 0.2 |  | 2.4 | ± | 0.3 |  |
| Xyl | 21.1 | ± | 1.4 |  | 18.5 | ± | 2.1 |  | 20.3 | ± | 1.9 |  |
| GalA | 3.6 | ± | 0.4 |  | 6.5 | ± | 0.4 | ** | 5.3 | ± | 1.1 |  |
| GlcA | 1.0 | ± | 0.1 |  | 1.4 | ± | 0.2 |  | 1.2 | ± | 0.1 |  |


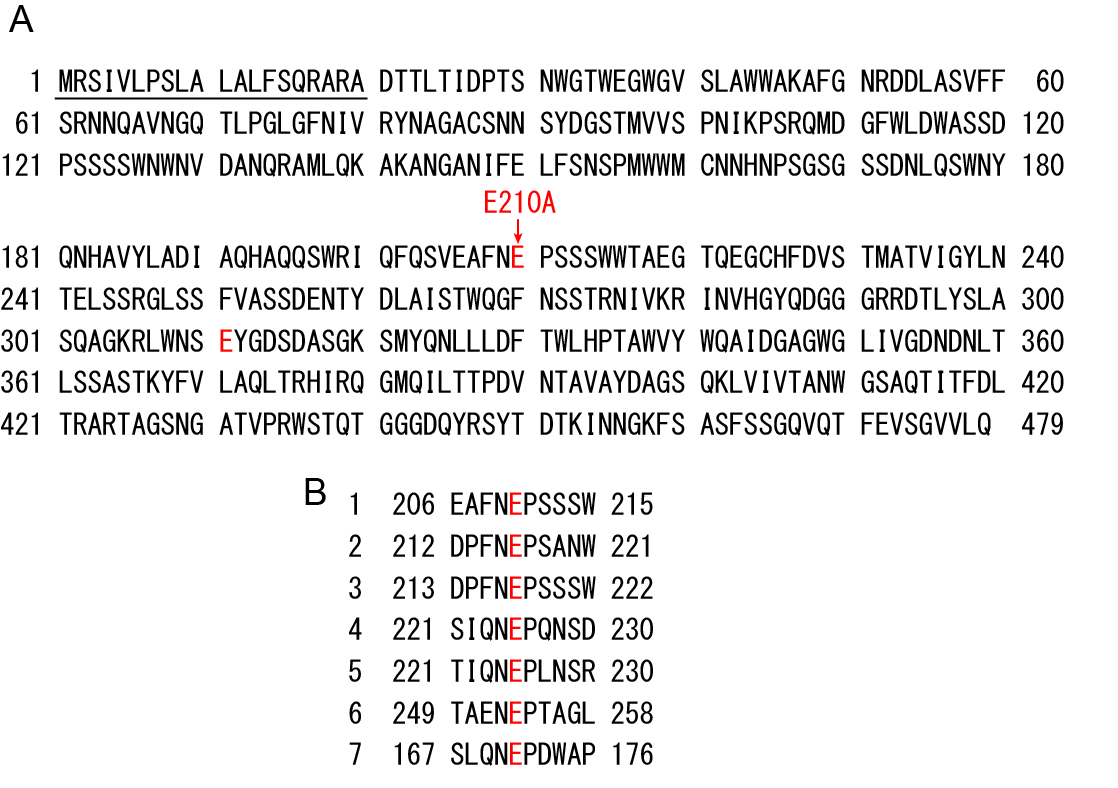


Supplementary Figure S1. Point mutation, E210A, introduced in Tv6GAL-PM. (A) Amino acid sequence of Tv6GAL. Tv6GAL has putative catalytic residues, E210 and E311, which are shown in red. In Tv6GAL-PM, the E210A mutation is introduced. The signal peptide is underlined. (B) Glu residue conserved in the GH 30 family. A partial amino acid sequence around E210 in Tv6GAL was aligned with those from other enzymes in GH 30 using the Clustal W program. 1, Tv6GAL; 2, endo-β-1,6-galactanase from *Neurospora crassa* OR74A (XP_330352.1); 3, endo-β-1,6-galactanase from *Streptomyces avermitilis* (BAC72917.1); 4, β-1,6-glucanase Neg1 from *N. crassa* (CAF06053.1); 5, glucan endo-β-1,6-glucosidase from *Lentinula edodes* (BAK52530.1); 6, glucocerebrosidase precursor from *Mus musculus* (AAA37671.1); 7, xylan degradation enzyme from *Clostridium acetobutylicum* ATCC 824 (AAK76864.1).


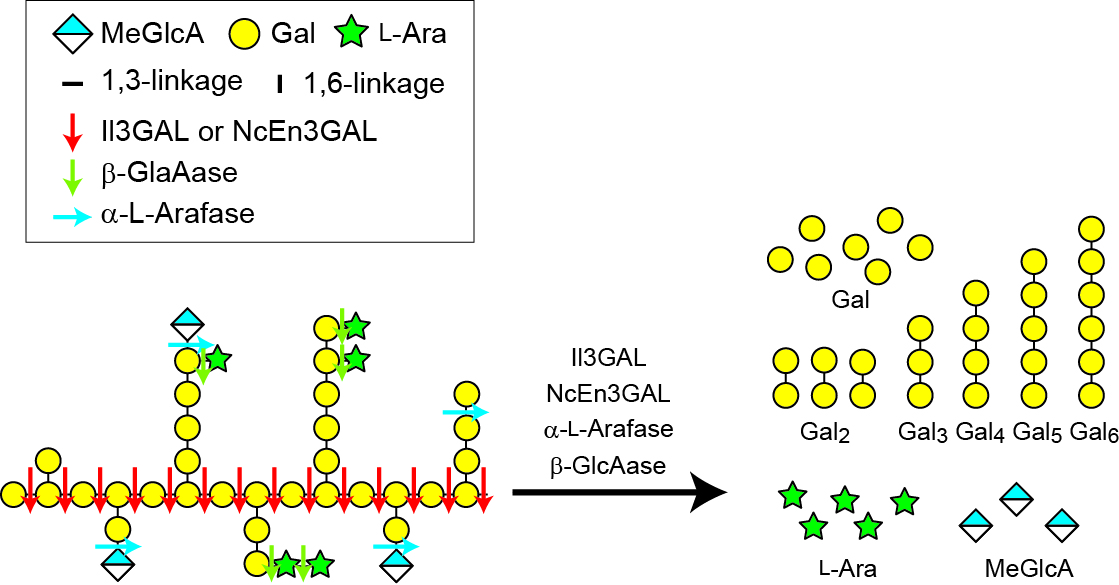


Supplementary Figure S2. Specific hydrolysis of type II AGs into β-1,6-galactooligosaccharides. Type II AGs included in the soluble fraction were hydrolyzed into β-1,6-galactooligosaccharides with exo-β-1,3-galactanase Il3GAL and endo-β-1,3-galactanase NcEn3GAL, an α-L-arabinofuranosidase from *Aspergillus niger* (α-L-Arafase) and a β-glucuronidase from *A. niger* (β-GlcAase). The released mono- and oligosaccharides were derivatized with ABEE and analyzed on HPLC. Note that released β-1,6-galactooligosaccharides respectively have one Gal residue derived from the β-1,3-galactan main chain. For example, β-1,6-Gal_3_ released in the hydrolysis corresponds to a β-1,6-Gal_2_ side chain in Figure 4.


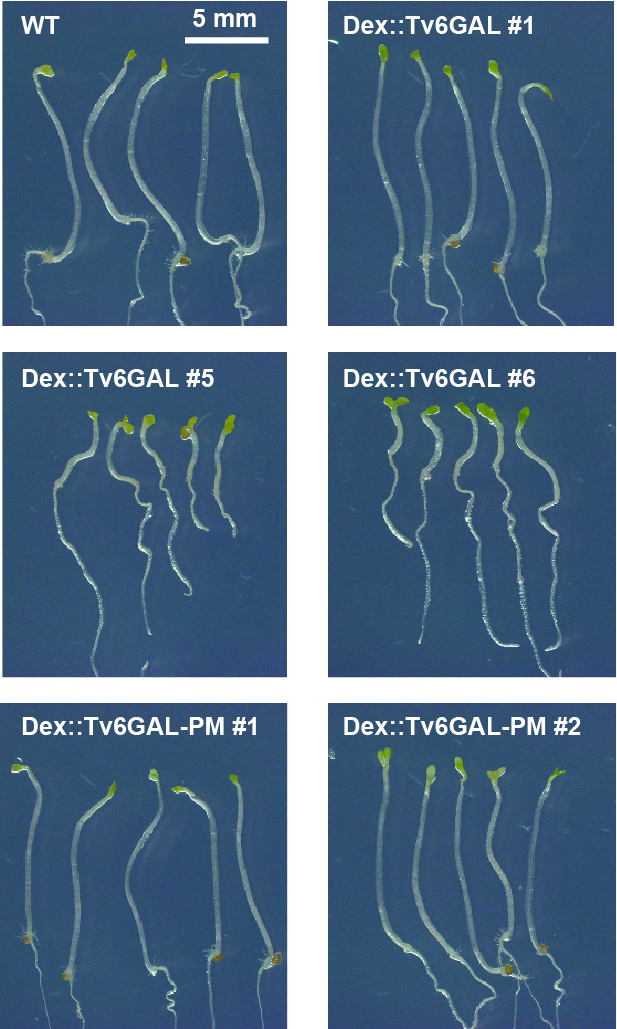


Supplementary Figure S3. Phenotype of dark-grown *Dex::Tv6GAL* plants. Plants were germinated and grown on MS-agar plate containing 10 µM Dex in the dark for 5 days. Representative hypocotyls are shown here. The bar graph for hypocotyl length is shown in Figure 5A. Plants were grown on MS-agar media containing 10 µM Dex under continuous light for 2 weeks.


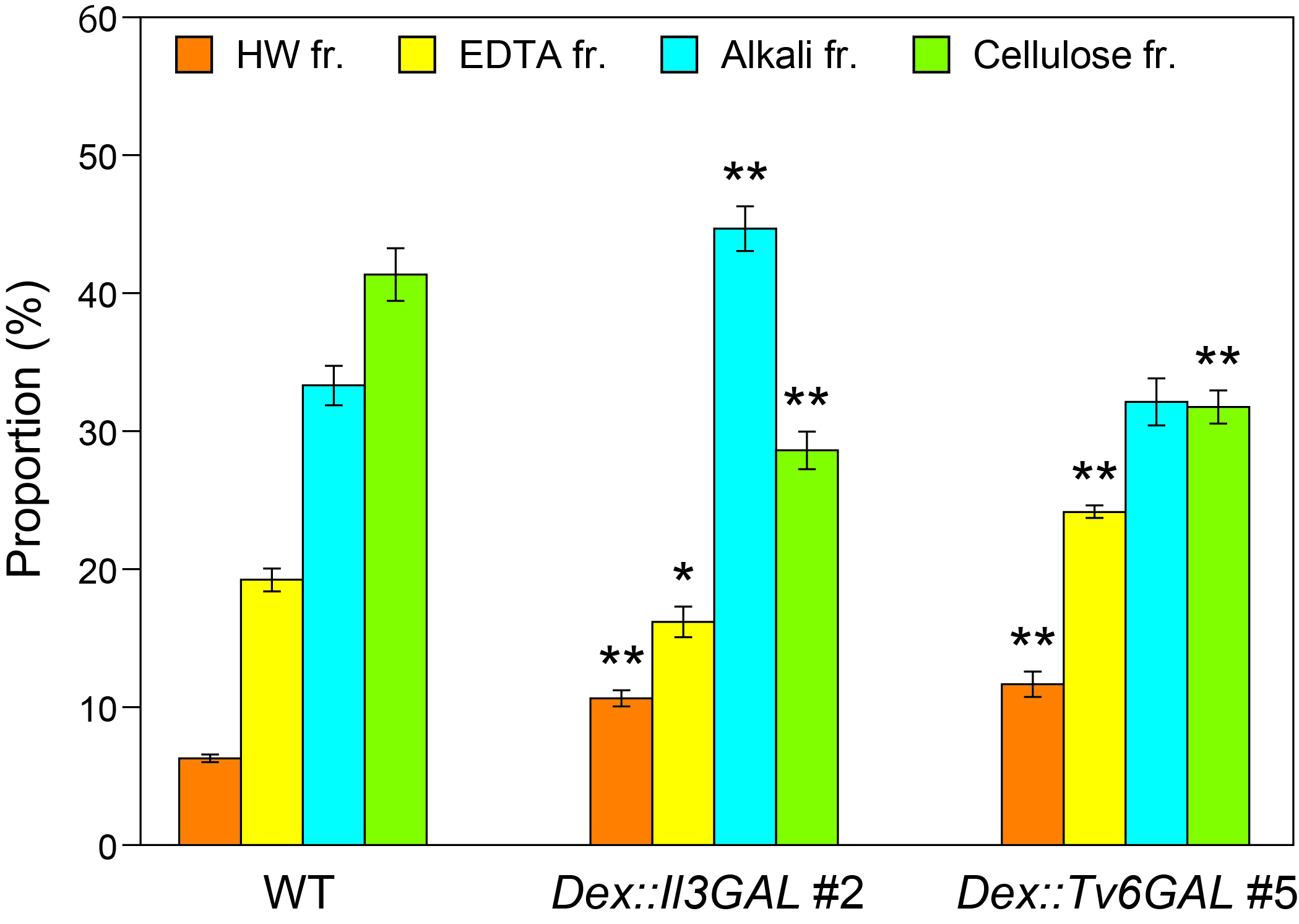


Supplementary Figure S4. Proportion of cell wall fractions. The seedlings grown with 10 µM Dex under continuous light for 10 days were homogenized in water. The homogenate was centrifuged and the supernatant was collected as the soluble fraction. The precipitate was heat-inactivated by boiling with 80% ethanol for 1 min, treated with α-amylase (20 units, porcine pancreatic, Sigma) at 37°C for 2 h, and centrifuged to remove starch. The sequential extractions with water, 50 mM EDTA (pH 6.8), 4 M potassium hydroxide containing 0.04% (w/v) sodium tetrahydroborate at 100°C were performed to obtain the hot water (HW), EDTA and alkali fractions, respectively. The residual precipitate was washed with water, ethanol and diethyl ether, and collected as the cellulose fraction. The cellulose fraction was hydrolyzed with 72% (v/v) sulfuric acid at 4°C for one hour and then 8% (v/v) sulfuric acid at 100°C for 4 hours. The total sugar amount of the fractions was measured by the phenol-sulfuric acid method (Dubois et al., 1956). Data are mean values with ±SD (n = 3 biological replicates). Asterisks indicate significant differences from WT plants (Student’s t-test, *, P<0.05; **, P<0.01). The line #5 of *Dex::Tv6GAL* plants had reduced proportion of cellulose, which was also observed for the line #2 of *Dex::Il3GAL* plants.
